# Percutaneous closure of a patent foramen ovale after cryptogenic stroke

**DOI:** 10.1007/s12471-017-1063-3

**Published:** 2017-12-04

**Authors:** R. J. R. Snijder, M. J. Suttorp, J. M. ten Berg, M. C. Post

**Affiliations:** Department of Cardiology, St. Antonius Hospital, Nieuwegein, The Netherlands

**Keywords:** Patent foramen ovale, Percutaneous PFO closure, Stroke

## Abstract

A patent foramen ovale is a common intracardiac finding that is located between the left and right atrium. It can cause right-to-left shunting and has a high prevalence in patients who suffer a cryptogenic stroke. Earlier trials did not show superiority of percutaneous patent foramen ovale closure with standard medical therapy over standard medical therapy alone in the treatment of cryptogenic stroke. Interestingly, several meta-analyses show positive results regarding closure, suggesting underpowering of the individual trials. Recently, two large prospective trials and one long-term follow-up study showed benefit of percutaneous closure over standard medical therapy in treatment of cryptogenic stroke. A larger right-to-left shunt or the presence of an atrial septal aneurysm were predictors for a recurrent event. Therefore, percutaneous patent foramen ovale closure after cryptogenic stroke should be recommended over antiplatelet therapy alone in patients younger than 55 years of age with a high-risk patent foramen ovale.

## Introduction

Patent foramen ovale (PFO) is a common intracardiac finding that is located between the left and right atrium and is found in about 25% of the population. In patients with cryptogenic stroke or transient ischaemic attack (TIA) the prevalence rises to 50% [1].

Large case-control studies showed an association between PFO and cryptogenic stroke, especially in patients younger than 55 years of age [2]. Several observational studies described a reduction in recurrent neurological events after percutaneous PFO closure with standard medical therapy (later mentioned as percutaneous PFO closure) compared with lifelong medical therapy [3, 4]. A few years ago, three prospective randomised trials failed to show superiority of percutaneous PFO closure. Long-term follow-up was lacking though. These trials differ in patient selection and type of device used [5–7]. However, several meta-analyses showed a benefit of percutaneous PFO closure suggesting underpowering of the individual studies [8, 9]. Recently, two large randomised controlled trials and one long-term follow-up study of an earlier trial were published. We discuss these trials in this point of view article and give our recommendation about the optimal treatment for cryptogenic stroke in the presence of a PFO.

## Earlier trials: clinical outcome and predictors for recurrence of stroke

Three prospective randomised trials have been published studying the difference between medical therapy and percutaneous PFO closure in patients who suffered from a cryptogenic stroke or TIA. A summary of these trials is shown in Tab. 1.

**Table 1:** Study characteristics of the CLOSURE-1, PC, RESPECT, Gore REDUCE and CLOSE trials

	CLOSURE-1	PC	RESPECT	Gore REDUCE	CLOSE
Patients (*n*)	909	414	980	664	663
Mean age (years)	45.9 ± 9.5	44.5 ± 10.1	45.9 ± 9.9	45.2 ± 9.4	43.3 ± 10.4
Male (%)	51.8	49.8	54.7	60.1	58.9
Moderate or large RLS (%)	52.9	65.6	75.2	81.3	100.0
ASA (%)	36.6	23.7	35.6	20.4^a^	32.8
*Treatment*					
Type of medical therapy	Aspirin, OAC or both	Antiplatelet, OAC or both	Antiplatelet or OAC	Antiplatelet	Antiplatelet or OAC
Oral anticoagulation (%)	34.0	31.0	25.0	0.0	28.2
Type of closure device	STARFlex	Amplatzer PFO	Amplatzer PFO	Helex septal occluder/Cardioform septal occluder	Any ICC-approved device
*Follow-up*					
Mean follow-up time (months)	44.0	49.0	31.0	38.4	63.6
Effective closure (%)	86.1	95.9	93.5	75.6	93.0
Drop-out medical therapy (%)	0.87	15.2	17.2	14.8	5.1
Drop-out closure device (%)	10.1	3.9	9.2	8.8	8.8
*Adverse events medical therapy*					
Major bleeding (%)	1.1	1.4	1.9	2.7	2.1
Atrial fibrillation (%)	0.7	1.0	1.5	0.4	0.9
*Adverse events closure device*					
Major procedural complication (%)	3.2	1.5	0.6	2.5	5.9
Non-procedural major bleeding (%)	2.6	0.5	1.6	0.9	0.8
Atrial fibrillation (%)	5.7	2.9	3.0	6.6	4.6
*Endpoints medical therapy*					
Stroke (%)	3.1	2.4	3.3	5.4	6.0
TIA (%)	4.1	3.3	–	–	–
Death (%)	0.0	0.0	1.2	0.0	0.0
*Endpoints closure device*					
Stroke (%)	2.9	0.5	1.8	1.4	0.0
TIA (%)	3.1	2.5	–	–	–
Death (%)	0.0	1.0	0.6	0.5	0
**Conclusion**	No significant benefit for closure	No significant benefit for closure	No significant benefit for closure	Significant benefit for closure	Significant benefit for closure

*ASA* atrial septal aneurysm, *ICC* Interventional Cardiology Committee,* OAC* oral anticoagulation, *PFO* patent foramen ovale, *RLS* right-to-left-shunt, *TIA* transient ischaemic attack

^a^ASA was only measured in CD group

The first trial, CLOSURE-1 (‘Evaluation of the STARFlex Septal Closure System in Patients with a Stroke and/or Transient Ischemic Attack due to Presumed Paradoxical Embolism through a Patent Foramen Ovale’), was published in 2012 and included 909 patients who presented with a cryptogenic stroke or TIA. At least a moderate right-to-left shunt (RLS) or an atrial septal aneurysm (ASA) was present in 52.9% and 36.6%, respectively. The STARFlex device (NMT Medicals) plus antiplatelet therapy (clopidogrel for 6 months and aspirin for 2 years) were used in the closure group and oral anticoagulation (OAC), aspirin or both in the medical therapy group at the discretion of the principal investigator. The primary endpoint, the composite of stroke/TIA during 2‑year follow-up, death from any cause during the first 30 days, and death from neurologic cause between 31 days and 2 years, was reached in 5.5% after closure and in 6.8% in the medical therapy group (adjusted hazard ratio (HR), 0.78; 95% confidence interval (CI), 0.45–1.35; *p* = 0.37). Subgroup analysis showed no predictors for recurrent stroke/TIA [5].

The PC trial (‘Using the Amplatzer PFO Occluder with Medical Treatment in Patients with Cryptogenic Embolism’) was published in 2013 and included 414 patients who suffered a cryptogenic stroke, TIA or a peripheral thromboembolic event. At least a moderate RLS was present in 65.6% and an ASA in 23.7%. The closure group received an Amplatzer device (St. Jude Medical), clopidogrel for 1–6 months and aspirin for at least 6 months. Medical therapy consisted of antiplatelet therapy, OAC, or both, at the discretion of the treating physician. During mean follow-up of 4.0 years, the primary endpoint (composite of death, non-fatal stroke, TIA or peripheral embolism) occurred after closure and after medical therapy, in 3.4% and 5.2%, respectively (HR 0.63, 95% CI: 0.24–1.62; *p* = 0.34). Subgroup analysis found no predictors for recurrent stroke/TIA [6].

Finally, the RESPECT trial (‘Randomised Evaluation of Recurrent Stroke Comparing PFO Closure to Established Current Standard of Care Treatment’), published in 2013, randomised 980 patients with cryptogenic stroke to closure (Amplatzer device, St. Jude Medical, plus aspirin and clopidogrel for 1 month, followed by aspirin alone for 5 months) or to medical therapy (aspirin, clopidogrel or warfarin). At least a moderate RLS was present in 75.2% and an ASA in 35.6%. After a mean follow-up of 2.6 years, the primary endpoint (composite of recurrent fatal and non-fatal ischaemic stroke, death from any cause within 30 days after implantation or 45 days after randomisation) was reached in the closure and medical therapy group in 3.4% and 5.2%, respectively (HR 0.49, 95% CI: 0.22–1.11; *p* = 0.08). Subgroup analysis showed a benefit for closure in presence of larger RLS (HR 0.18, 95% CI: 0.04–0.81; *p* = 0.01) or ASA (HR 0.19, 95% CI: 0.04–0.87; *p* = 0.02) [7].

None of these three trials showed superiority of percutaneous closure over medical therapy. However, the trials had study limitations and are difficult to compare. Firstly, the dropout rate was high, based on crossover to closure. Secondly, different devices were used for percutaneous closure. The CLOSURE-1 trial used the STARFlex device, which is already off the market due to poorer efficacy and safety data. Both the PC and the RESPECT trial used the Amplatzer device, which has proven to be safe and effective. Thirdly, different primary endpoints were used: the CLOSURE-1 and PC trials used stroke and TIA as endpoint, the RESPECT trial only stroke. Patient selection was different between trials. For instance, the CLOSURE-1 trial included patients without a proven stroke on imaging, where the PC trial also included patients with a peripheral embolism. Long-term medical therapy was different between trials as well, based on physicians’ preference. Finally, all trials had a modest statistical power with a relatively small population and low clinical event rates.

Even though PFO closure was at least equal in comparison to medical therapy, it did not change the guidelines or clinical practice.

## Meta-analyses and review

Several meta-analyses were published discussing these trials mentioned above, including a total of 2,303 patients.

Khan et al. suggest that PFO closure is beneficial when compared with medical therapy in the prevention of recurrent stroke. The effect-estimate HR was 0.67 (95% CI: 0.44–1.00; *p* = 0.05) in the intention-to-treat, 0.62 (95% CI: 0.40–0.95; *p* = 0.03) using per-protocol, and 0.61 (95% CI: 0.40–0.95; *p* = 0.03) using the as-treated cohort, all showing a beneficial effect for PFO closure. After pooling the results of the trials using the Amplatzer device, the results showed an even more positive effect for PFO closure (HR 0.54, 95% CI: 0.29–1.01; *p* = 0.05) [8].

Rengifo-Moreno et al. defined primary outcome as a recurrent stroke and/or TIA and found a significant risk reduction after PFO closure (pooled HR 0.59, 95% CI: 0.36–0.97; *p* = 0.04). The composite outcome of death, neurological events and peripheral embolism based on intention-to-treat analyses showed a possible benefit for closure (pooled HR 0.67, 95% CI: 0.44–1.00; *p* = 0.05). A substantial RLS at baseline tended to be associated with a decrease in vascular events after closure (pooled HR 0.35, 95% CI: 0.12–1.03; *p* = 0.06) [9].

There were also meta-analyses without a statistically significant difference between closure and medical therapy. Ntaios et al. and Kwong et al. showed no significant difference in recurrent stroke between closure and medical therapy (odds ratio (OR) 0.64, *p* = 0.11 and OR 0.65, *p* = 0.17, respectively). Furthermore, there was no difference in the occurrence of TIA or death between both treatment arms. Subgroup analyses showed a possible benefit on stroke rate for percutaneous closure using the Amplatzer PFO occluder (OR 0.46, *p* = 0.04 and OR 0.47, *p* = 0.06, respectively) [10, 11]. And finally, a review by Li et al. showed no statistically significant difference in recurrent stroke or TIA between closure and medical therapy (relatively risk 0.73; 95% CI: 0.45–1.17) [12].

The meta-analyses described above showed similar complications, mainly vascular complications and atrial fibrillation. The complications were significantly higher after closure.

## Recent trials

Recently, two large randomised trials and one long-term follow-up study of the earlier described RESPECT trial were published [13–15]. A summary of these trials is shown in Tab. 1.

The Gore REDUCE trial (‘GORE HELEX Septal Occluder/GORE CARDIOFORM Septal Occluder for Patent Foramen Ovale Closure in Stroke Patients’) randomised 664 patients in a 2:1 ratio to percutaneous closure (Helex septal occluder/Cardioform septal occluder, W.L. Gore and Associates) plus antiplatelet therapy or antiplatelet therapy alone. The co-primary endpoints were freedom from clinical evidence of ischaemic stroke and incidence of new brain infarction, which was a composite of clinical ischaemic stroke or silent brain infarction detected on imaging, both 24 months after randomisation. At least a moderate RLS was present in 81.3% and an ASA in 20.4%. During a median follow-up of 3.2 years, stroke occurred after closure and after medical therapy in 1.4% and 5.4%, respectively (HR 0.23; 95% CI: 0.09–0.62; *p* = 0.002). New brain infarctions were found in 5.7% and 11.3%, respectively (HR 0.51; 95% CI: 0.29–0.91; *p* = 0.04). A significant benefit for closure was found in patients having a substantial RLS (HR 0.18; 95% CI: 0.06–0.58; *p* = 0.001). Atrial fibrillation occurred significantly more often in the closure group (*p* < 0.001). However, there was no significant difference in the overall serious adverse events (*p* = 0.22) [13].

The CLOSE trial (‘Patent Foramen Ovale Closure or Anticoagulants Versus Antiplatelet Therapy to Prevent Stroke Recurrence’) assigned 663 patients who recently suffered a stroke in the presence of high-risk PFO (large RLS or ASA) in a 1:1:1 ratio to percutaneous PFO closure plus long-term antiplatelet therapy, antiplatelet therapy alone or OAC alone. At least a moderate RLS was present in 100% and an ASA in 32.8%. During a mean follow-up of 5.3 years, the primary endpoint (occurrence of stroke) was reached in 0% in the closure group, in 6.0% in the antiplatelet group (HR 0.03; 95% CI: 0–0.26; *p* = <0.001), and 1.6% in the OAC group. This study was not powered to compare the outcome between antiplatelet therapy and OAC. Closure-related events occurred in 5.9%. There was no significant difference in serious adverse events between both treatment arms (*p* = 0.56). Onset of atrial fibrillation occurred significantly more often after closure (*p* = 0.02) [14].

The previously described RESPECT trial published data with an extended median follow-up of 5.9 years. The number of patients and the primary endpoint are described above. The two groups were not equal at the latest follow-up due to a high dropout rate (33.3%) in the medically treated group. Recurrent non-fatal stroke occurred in 3.6% (0.58 events per 100 patient-years) after closure and in 5.8% (1.07 events per 100 patient-years) receiving medical therapy (HR 0.55; 95% CI: 0.31–0.999; *p* = 0.046). Subgroup analysis showed a benefit for closure in presence of a substantial RLS (HR 0.26; 95% CI: 0.10–0.71; *p* = 0.005) or ASA (HR 0.20; 95% CI: 0.06–0.70; *p* = 0.005). Both serious adverse events and atrial fibrillation did not significantly differ between both groups [15].

In summary, the two recent trials and the extended follow-up study showed a significant benefit for percutaneous PFO closure when compared with medical therapy alone (especially antiplatelet therapy) in younger patients who suffer a cryptogenic stroke. This beneficial effect was greater in patients with a high-risk PFO (defined as a PFO with a at least a moderate RLS and/or ASA).

The difference between the earlier and recent trials could be explained by the fact that recent trials were more uniform, had a long-term follow-up and a different endpoint compared with earlier trials. More importantly, recent trials included patients with at least a moderate RLS and/or an ASA, which are known as high-risk PFOs, and showed an even greater effect of closure.

## How to decide which PFO to close?

In the past, literature did not reach consensus in which symptomatic patient group the PFO should be closed. The latest ESC guidelines describe a class IIa level of evidence C for percutaneous PFO closure in young patients with a systemic paradoxical embolism [16]. The 2017 Dutch guideline acknowledges the potential benefit for percutaneous PFO closure in selected patients; class IIa level of evidence A [17].

Age and presence of atherosclerosis play an important role in whether PFO is a likely cause of cryptogenic stroke. Pezzini et al. showed that with lesser risk factors for atherosclerosis, the influence of PFO increases [18]. Kent et al. created a calculator to stratify the likelihood of PFO related to stroke [19]. The Risk of Paradoxical Embolism (RoPE) calculator uses important risk factors for atherosclerosis (hypertension, diabetes, history of stroke or TIA and smoking), the presence of a cortical infarct on imaging and age for identifying a PFO related stroke. The higher the RoPE score (between 0 and 10 points), the more likely a PFO is related to stroke. Using a multivariate model after inclusion of more than 3,000 patients, younger age and the absence of risk factors mentioned above were found to be associated with the presence of a PFO related stroke. The optimal cut-off value to identify a stroke-related PFO was a RoPE score of at least 6. However, presence of a large shunt or ASA was not included even though previous studies have shown an association between cryptogenic stroke and these high-risk PFOs [20]. Using echocardiography, ASA is defined as more than 10 mm bulging of the atrial septum and the severity of a RLS is calculated on the maximum amount of bubbles in the left atrium, and graded as minimal, moderate or large [21, 22]. In contrast to the CLOSURE-1 and PC trial, the earlier RESPECT trial found a significant benefit for closure in presence of a large RLS or ASA. The more recent studies (Gore REDUCE and RESPECT) found a large RLS or ASA as predictors for benefit of PFO closure. Moreover, the CLOSE trial only included patients with these PFO characteristics and showed an overall significant, positive effect for closure. The difference in significance between the earlier and recent trials could be explained by the limitations of the earlier trials.

Therefore, after exclusion of other possible causes, percutaneous PFO closure using a safe and effective device in combination with medical therapy should be recommended over medical therapy alone in young patients (≤ 55 years of age) who suffer a cryptogenic stroke confirmed by cerebral imaging. A RoPE score of at least 6 or a high-risk (at least moderate RLS or ASA) PFO should be present. Adverse events of PFO closure should be taken into account. A recent study including more than 1,800 percutaneous PFO closure procedures showed a complication rate of 4.9% in patients younger than 60 years of age (mainly vascular complications and paroxysmal atrial fibrillation). This rate is similar when compared with the recent trials [23]**.**

**Fig. 1:**
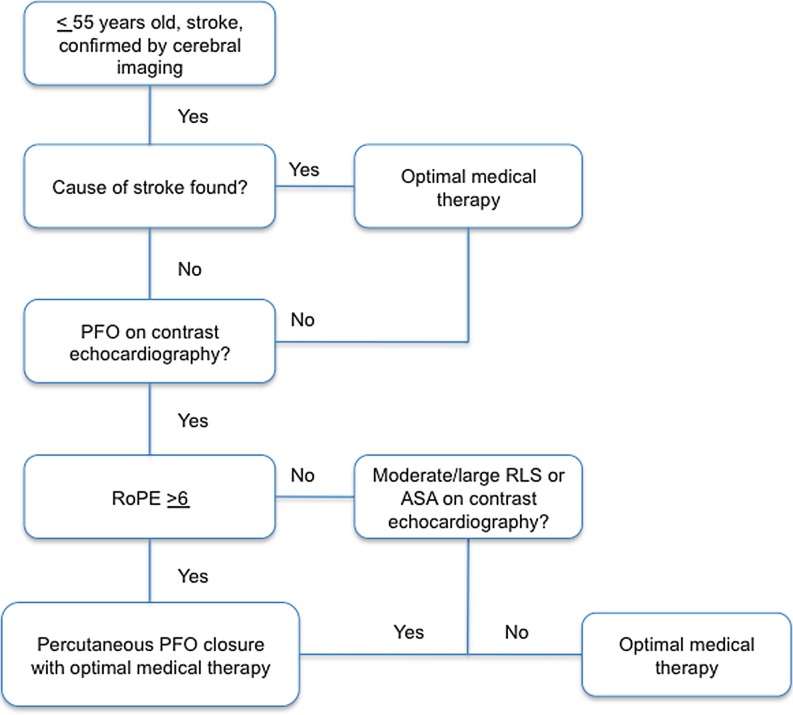
Algorithm for the treatment of cryptogenic stroke. *ASA* atrial septal aneurysm,* PFO* patent foramen ovale, *RLS* right-to-left shunt, *RoPE* risk of paradoxical embolism

In Fig. 1, we suggest an algorithm for the treatment of cryptogenic stroke in the presence of PFO.

## Recommendation

At this moment, literature shows a significant beneficial effect of percutaneous PFO closure over medical therapy alone in selected patients who suffer a cryptogenic stroke. It is important to exclude other possible causes of stroke before considering percutaneous closure. The RoPE score, described earlier, could be a useful instrument in determining the possible association between stroke and the presence of a PFO, but the score might underestimate the risk. It has become clear that the presence of a significant RLS or an ASA are important discriminators to determine whether a stroke is related to a PFO. Based on the currently available literature, the current guidelines should be updated in favour of percutaneous PFO closure in young patients who suffer a cryptogenic stroke. The RoPE score and the presence of a high-risk PFO should be important factors to guide the decision.

## Conclusion

Percutaneous PFO closure should be the recommended treatment over medical therapy in young patients suffering cryptogenic stroke.
